# (Methano­lato-κ*O*)[*N*′-(3-meth­oxy-2-oxidobenzyl­idene-κ*O* ^2^)-4-nitro­benzo­hydrazidato-κ^2^ *N*′,*O*]oxidovanadium(V)

**DOI:** 10.1107/S1600536811040207

**Published:** 2011-10-05

**Authors:** Chen-Yi Wang, Hai-Yu Tu, Pei-Fei Zhu, Su-Jun Sheng

**Affiliations:** a Department of Chemistry, Huzhou University, Huzhou 313000, People’s Republic of China

## Abstract

The title oxidovanadium(V) complex, [V(C_15_H_11_N_3_O_5_)(CH_3_O)O], was obtained by the reaction of 2-hy­droxy-3-meth­oxy­benzaldehyde, 4-nitro­benzohydrazide and vanadyl sulfate in methanol. The V^V^ atom is five-coordinated by the two O and one N donor atoms of the Schiff base ligand, one methano­late O atom and one oxido O atom, forming a distorted square-pyramidal geometry.

## Related literature

For Schiff base complexes, see: Wang (2009 ▶); Wang & Ye (2011 ▶). For similar oxidovanadium complexes, see: Deng *et al.* (2005 ▶); Gao *et al.* (2005 ▶); Huo *et al.* (2004 ▶).
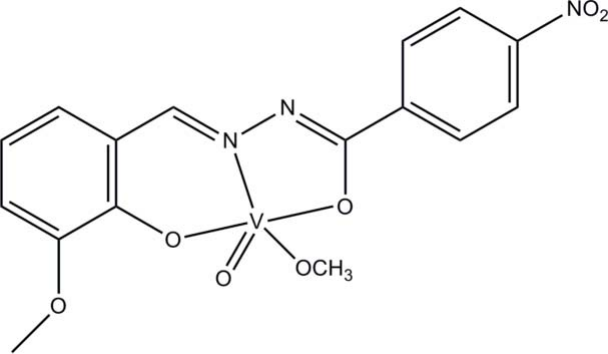


## Experimental

### 

#### Crystal data

[V(C_15_H_11_N_3_O_5_)(CH_3_O)O]
*M* *_r_* = 411.24
Triclinic, 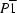

*a* = 6.410 (3) Å
*b* = 10.253 (3) Å
*c* = 13.490 (3) Å
α = 71.111 (2)°
β = 87.998 (2)°
γ = 86.473 (2)°
*V* = 837.2 (5) Å^3^
*Z* = 2
Mo *K*α radiation
μ = 0.64 mm^−1^
*T* = 298 K
0.20 × 0.20 × 0.20 mm

#### Data collection

Bruker SMART CCD area-detector diffractometer
Absorption correction: multi-scan (*SADABS*; Sheldrick, 1996 ▶) *T*_min_ = 0.883, *T*_max_ = 0.883
6739 measured reflections
3538 independent reflections
2686 reflections with *I* > 2σ(*I*)
*R*_int_ = 0.029

#### Refinement

*R*[*F* ^2^ > 2σ(*F* ^2^)] = 0.051
*wR*(*F* ^2^) = 0.115
*S* = 1.15
3538 reflections
246 parameters
H-atom parameters constrained
Δρ_max_ = 0.47 e Å^−3^
Δρ_min_ = −0.37 e Å^−3^

### 

Data collection: *SMART* (Bruker, 1998 ▶); cell refinement: *SAINT* (Bruker, 1998 ▶); data reduction: *SAINT*; program(s) used to solve structure: *SHELXS97* (Sheldrick, 2008 ▶); program(s) used to refine structure: *SHELXL97* (Sheldrick, 2008 ▶); molecular graphics: *SHELXTL* (Sheldrick, 2008 ▶); software used to prepare material for publication: *SHELXTL*.

## Supplementary Material

Crystal structure: contains datablock(s) global, I. DOI: 10.1107/S1600536811040207/qm2033sup1.cif

Structure factors: contains datablock(s) I. DOI: 10.1107/S1600536811040207/qm2033Isup2.hkl

Additional supplementary materials:  crystallographic information; 3D view; checkCIF report

## Figures and Tables

**Table 1:** Selected bond lengths (Å)

V1—O6	1.566 (2)
V1—O7	1.743 (2)
V1—O1	1.816 (2)
V1—O3	1.922 (2)
V1—N1	2.095 (3)

**Fig. 1.**
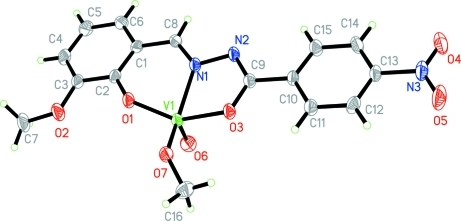
The molecular structure of the title compound, showing the atom-numbering scheme. Displacement ellipsoids are drawn at the 30% probability level.

## supplementary crystallographic information

### Comment

As part of our investigations into new Schiff base complexes (Wang & Ye, 2011; Wang, 2009), we have synthesized the title compound, a new mononuclear oxovanadium(V) complex, Fig. 1. The V atom in the complex is five-coordinated by the NNO donor atoms of the Schiff base ligand, one methoxy O atom, and one oxo O atom, forming a square pyramidal geometry. The V–O and V–N bond lengths (Table 1) are typical and are comparable with those observed in other similar vanadium complexes (Deng *et al.*, 2005; Gao *et al.*, 2005; Huo *et al.*, 2004).

### Experimental

2-Hydroxy-3-methoxybenzaldehyde (1.0 mmol, 0.15 g), 4-nitrobenzohydrazide (1.0 mmol, 0.18 g), and vanadyl sulfate (1.0 mmol, 0.16 g) were dissolved in methanol (30 ml). The mixture was stirred at room temperature for 10 min to give a clear brown solution. After keeping the solution in air for a week, brown block-shaped crystals were formed at the bottom of the vessel.

### Refinement

All H atoms were placed in geometrically idealized positions and constrained to ride on their parent atoms, with C—H distances in the range 0.93–0.96 Å, and with *U*_iso_(H) set at 1.2 or 1.5*U*_eq_(C).

### Crystal data

**Table:**

[V(C_15_H_11_N_3_O_5_)(CH_3_O)O]	*Z* = 2
*M**_r_* = 411.24	*F*(000) = 420
Triclinic, *P*1	*D*_x_ = 1.631 Mg m^−^^3^
*a* = 6.410 (3) Å	Mo *K*α radiation, λ = 0.71073 Å
*b* = 10.253 (3) Å	Cell parameters from 2551 reflections
*c* = 13.490 (3) Å	θ = 3.0–28.2°
α = 71.111 (2)°	µ = 0.64 mm^−^^1^
β = 87.998 (2)°	*T* = 298 K
γ = 86.473 (2)°	Block, brown
*V* = 837.2 (5) Å^3^	0.20 × 0.20 × 0.20 mm

### Data collection

**Table:**

Bruker SMART CCD area-detector diffractometer	3538 independent reflections
Radiation source: fine-focus sealed tube	2686 reflections with *I* > 2σ(*I*)
graphite	*R*_int_ = 0.029
ω scans	θ_max_ = 27.0°, θ_min_ = 2.2°
Absorption correction: multi-scan (*SADABS*; Sheldrick, 1996)	*h* = −8→8
*T*_min_ = 0.883, *T*_max_ = 0.883	*k* = −13→13
6739 measured reflections	*l* = −17→16

### Refinement

**Table:**

Refinement on *F*^2^	Primary atom site location: structure-invariant direct methods
Least-squares matrix: full	Secondary atom site location: difference Fourier map
*R*[*F*^2^ > 2σ(*F*^2^)] = 0.051	Hydrogen site location: inferred from neighbouring sites
*wR*(*F*^2^) = 0.115	H-atom parameters constrained
*S* = 1.15	*w* = 1/[σ^2^(*F*_o_^2^) + (0.0313*P*)^2^ + 0.6271*P*] where *P* = (*F*_o_^2^ + 2*F*_c_^2^)/3
3538 reflections	(Δ/σ)_max_ < 0.001
246 parameters	Δρ_max_ = 0.47 e Å^−^^3^
0 restraints	Δρ_min_ = −0.37 e Å^−^^3^

### Special details

**Table:**

Geometry. All e.s.d.'s (except the e.s.d. in the dihedral angle between two l.s. planes) are estimated using the full covariance matrix. The cell e.s.d.'s are taken into account individually in the estimation of e.s.d.'s in distances, angles and torsion angles; correlations between e.s.d.'s in cell parameters are only used when they are defined by crystal symmetry. An approximate (isotropic) treatment of cell e.s.d.'s is used for estimating e.s.d.'s involving l.s. planes.
Refinement. Refinement of *F*^2^ against ALL reflections. The weighted *R*-factor *wR* and goodness of fit *S* are based on *F*^2^, conventional *R*-factors *R* are based on *F*, with *F* set to zero for negative *F*^2^. The threshold expression of *F*^2^ > σ(*F*^2^) is used only for calculating *R*-factors(gt) *etc*. and is not relevant to the choice of reflections for refinement. *R*-factors based on *F*^2^ are statistically about twice as large as those based on *F*, and *R*- factors based on ALL data will be even larger.

### Fractional atomic coordinates and isotropic or equivalent isotropic displacement parameters (Å^2^)

**Table:**

	*x*	*y*	*z*	*U*_iso_*/*U*_eq_	
V1	0.33384 (8)	0.75890 (6)	0.64666 (4)	0.03741 (18)	
N1	0.2573 (4)	0.7590 (2)	0.7989 (2)	0.0367 (6)	
N2	0.3794 (4)	0.8370 (3)	0.8399 (2)	0.0400 (6)	
N3	1.1433 (5)	1.2126 (3)	0.8496 (3)	0.0533 (8)	
O1	0.1727 (3)	0.6105 (2)	0.68310 (17)	0.0450 (6)	
O2	−0.0923 (4)	0.4642 (3)	0.62674 (19)	0.0571 (7)	
O3	0.5567 (3)	0.8465 (2)	0.68799 (17)	0.0438 (6)	
O4	1.1239 (4)	1.2504 (3)	0.9257 (2)	0.0785 (9)	
O5	1.2866 (4)	1.2421 (3)	0.7875 (3)	0.0847 (10)	
O6	0.1922 (4)	0.8862 (2)	0.58276 (19)	0.0564 (6)	
O7	0.5102 (3)	0.7131 (2)	0.56069 (17)	0.0469 (6)	
C1	−0.0354 (5)	0.6150 (3)	0.8311 (2)	0.0376 (7)	
C2	0.0009 (5)	0.5759 (3)	0.7423 (2)	0.0372 (7)	
C3	−0.1420 (5)	0.4947 (3)	0.7153 (3)	0.0413 (8)	
C4	−0.3148 (5)	0.4535 (3)	0.7785 (3)	0.0497 (9)	
H4	−0.4103	0.4001	0.7610	0.060*	
C5	−0.3481 (5)	0.4906 (4)	0.8679 (3)	0.0541 (9)	
H5	−0.4649	0.4610	0.9102	0.065*	
C6	−0.2115 (5)	0.5703 (3)	0.8950 (3)	0.0480 (9)	
H6	−0.2353	0.5947	0.9552	0.058*	
C7	−0.2465 (6)	0.4031 (4)	0.5845 (3)	0.0626 (11)	
H7A	−0.2655	0.3107	0.6301	0.094*	
H7B	−0.2013	0.4008	0.5165	0.094*	
H7C	−0.3764	0.4566	0.5787	0.094*	
C8	0.1051 (5)	0.7005 (3)	0.8586 (2)	0.0388 (7)	
H8	0.0856	0.7146	0.9231	0.047*	
C9	0.5315 (5)	0.8793 (3)	0.7736 (2)	0.0370 (7)	
C10	0.6881 (4)	0.9669 (3)	0.7941 (2)	0.0364 (7)	
C11	0.8536 (5)	1.0068 (3)	0.7247 (3)	0.0452 (8)	
H11	0.8644	0.9799	0.6651	0.054*	
C12	1.0030 (5)	1.0865 (3)	0.7439 (3)	0.0482 (9)	
H12	1.1167	1.1119	0.6984	0.058*	
C13	0.9818 (5)	1.1275 (3)	0.8302 (3)	0.0400 (8)	
C14	0.8183 (5)	1.0912 (4)	0.8996 (3)	0.0532 (9)	
H14	0.8062	1.1209	0.9579	0.064*	
C15	0.6721 (5)	1.0095 (4)	0.8809 (3)	0.0522 (9)	
H15	0.5607	0.9827	0.9278	0.063*	
C16	0.6484 (6)	0.7807 (4)	0.4806 (3)	0.0659 (11)	
H16A	0.5745	0.8560	0.4301	0.099*	
H16B	0.7069	0.7170	0.4471	0.099*	
H16C	0.7586	0.8156	0.5097	0.099*	

### Atomic displacement parameters (Å^2^)

**Table:**

	*U*^11^	*U*^22^	*U*^33^	*U*^12^	*U*^13^	*U*^23^
V1	0.0382 (3)	0.0428 (3)	0.0366 (3)	−0.0148 (2)	0.0045 (2)	−0.0186 (2)
N1	0.0344 (14)	0.0391 (14)	0.0411 (16)	−0.0128 (11)	0.0014 (12)	−0.0175 (12)
N2	0.0377 (14)	0.0468 (15)	0.0426 (16)	−0.0183 (12)	0.0045 (12)	−0.0219 (13)
N3	0.0438 (18)	0.0463 (17)	0.071 (2)	−0.0160 (14)	−0.0103 (16)	−0.0175 (16)
O1	0.0440 (13)	0.0521 (13)	0.0486 (14)	−0.0239 (10)	0.0104 (11)	−0.0271 (11)
O2	0.0540 (15)	0.0696 (16)	0.0620 (17)	−0.0260 (12)	−0.0003 (12)	−0.0370 (14)
O3	0.0393 (12)	0.0557 (14)	0.0466 (14)	−0.0223 (10)	0.0103 (10)	−0.0280 (11)
O4	0.079 (2)	0.095 (2)	0.080 (2)	−0.0395 (17)	−0.0057 (16)	−0.0476 (18)
O5	0.0576 (17)	0.104 (2)	0.107 (2)	−0.0502 (17)	0.0142 (17)	−0.048 (2)
O6	0.0608 (15)	0.0550 (15)	0.0543 (16)	−0.0038 (12)	−0.0042 (12)	−0.0183 (12)
O7	0.0485 (13)	0.0544 (14)	0.0464 (14)	−0.0212 (11)	0.0156 (11)	−0.0264 (11)
C1	0.0349 (17)	0.0403 (17)	0.0357 (18)	−0.0124 (13)	0.0003 (14)	−0.0077 (14)
C2	0.0336 (17)	0.0352 (16)	0.0416 (19)	−0.0111 (13)	−0.0011 (14)	−0.0092 (14)
C3	0.0410 (18)	0.0393 (17)	0.044 (2)	−0.0108 (14)	−0.0085 (15)	−0.0113 (15)
C4	0.0394 (19)	0.049 (2)	0.060 (2)	−0.0207 (15)	−0.0070 (17)	−0.0134 (18)
C5	0.0399 (19)	0.064 (2)	0.057 (2)	−0.0249 (17)	0.0075 (17)	−0.0141 (19)
C6	0.047 (2)	0.057 (2)	0.041 (2)	−0.0201 (16)	0.0067 (16)	−0.0144 (17)
C7	0.066 (2)	0.060 (2)	0.074 (3)	−0.0171 (19)	−0.020 (2)	−0.034 (2)
C8	0.0394 (18)	0.0453 (18)	0.0340 (18)	−0.0129 (14)	0.0038 (14)	−0.0145 (15)
C9	0.0347 (17)	0.0406 (17)	0.0398 (19)	−0.0091 (13)	0.0008 (14)	−0.0174 (15)
C10	0.0338 (16)	0.0357 (16)	0.0429 (19)	−0.0095 (13)	0.0006 (14)	−0.0159 (14)
C11	0.0415 (18)	0.052 (2)	0.050 (2)	−0.0153 (15)	0.0088 (16)	−0.0269 (17)
C12	0.0362 (18)	0.052 (2)	0.058 (2)	−0.0175 (15)	0.0131 (16)	−0.0183 (18)
C13	0.0322 (17)	0.0385 (17)	0.051 (2)	−0.0094 (13)	−0.0069 (15)	−0.0151 (15)
C14	0.054 (2)	0.069 (2)	0.051 (2)	−0.0239 (18)	0.0049 (17)	−0.0351 (19)
C15	0.045 (2)	0.072 (2)	0.051 (2)	−0.0265 (17)	0.0119 (17)	−0.0320 (19)
C16	0.069 (3)	0.069 (3)	0.063 (3)	−0.025 (2)	0.028 (2)	−0.023 (2)

### Geometric parameters (Å, °)

**Table:**

V1—O6	1.566 (2)	C4—H4	0.9300
V1—O7	1.743 (2)	C5—C6	1.368 (4)
V1—O1	1.816 (2)	C5—H5	0.9300
V1—O3	1.922 (2)	C6—H6	0.9300
V1—N1	2.095 (3)	C7—H7A	0.9600
N1—C8	1.290 (4)	C7—H7B	0.9600
N1—N2	1.397 (3)	C7—H7C	0.9600
N2—C9	1.295 (4)	C8—H8	0.9300
N3—O5	1.206 (4)	C9—C10	1.477 (4)
N3—O4	1.209 (4)	C10—C15	1.375 (4)
N3—C13	1.476 (4)	C10—C11	1.380 (4)
O1—C2	1.334 (3)	C11—C12	1.380 (4)
O2—C3	1.353 (4)	C11—H11	0.9300
O2—C7	1.428 (4)	C12—C13	1.360 (4)
O3—C9	1.306 (3)	C12—H12	0.9300
O7—C16	1.398 (4)	C13—C14	1.367 (4)
C1—C2	1.390 (4)	C14—C15	1.376 (4)
C1—C6	1.402 (4)	C14—H14	0.9300
C1—C8	1.431 (4)	C15—H15	0.9300
C2—C3	1.406 (4)	C16—H16A	0.9600
C3—C4	1.375 (4)	C16—H16B	0.9600
C4—C5	1.383 (5)	C16—H16C	0.9600
			
O6—V1—O7	108.77 (12)	C1—C6—H6	120.3
O6—V1—O1	106.79 (12)	O2—C7—H7A	109.5
O7—V1—O1	99.86 (10)	O2—C7—H7B	109.5
O6—V1—O3	101.83 (11)	H7A—C7—H7B	109.5
O7—V1—O3	88.11 (10)	O2—C7—H7C	109.5
O1—V1—O3	145.77 (10)	H7A—C7—H7C	109.5
O6—V1—N1	99.57 (12)	H7B—C7—H7C	109.5
O7—V1—N1	149.12 (11)	N1—C8—C1	123.6 (3)
O1—V1—N1	82.96 (9)	N1—C8—H8	118.2
O3—V1—N1	74.03 (9)	C1—C8—H8	118.2
C8—N1—N2	115.3 (2)	N2—C9—O3	122.9 (3)
C8—N1—V1	128.1 (2)	N2—C9—C10	120.1 (3)
N2—N1—V1	116.50 (18)	O3—C9—C10	117.0 (3)
C9—N2—N1	106.9 (2)	C15—C10—C11	119.4 (3)
O5—N3—O4	123.5 (3)	C15—C10—C9	121.3 (3)
O5—N3—C13	117.9 (3)	C11—C10—C9	119.3 (3)
O4—N3—C13	118.6 (3)	C12—C11—C10	119.9 (3)
C2—O1—V1	134.51 (19)	C12—C11—H11	120.1
C3—O2—C7	117.9 (3)	C10—C11—H11	120.1
C9—O3—V1	118.25 (18)	C13—C12—C11	119.2 (3)
C16—O7—V1	136.8 (2)	C13—C12—H12	120.4
C2—C1—C6	119.8 (3)	C11—C12—H12	120.4
C2—C1—C8	120.8 (3)	C12—C13—C14	122.2 (3)
C6—C1—C8	119.3 (3)	C12—C13—N3	118.4 (3)
O1—C2—C1	121.3 (2)	C14—C13—N3	119.3 (3)
O1—C2—C3	118.9 (3)	C13—C14—C15	118.2 (3)
C1—C2—C3	119.8 (3)	C13—C14—H14	120.9
O2—C3—C4	126.0 (3)	C15—C14—H14	120.9
O2—C3—C2	114.8 (3)	C10—C15—C14	121.0 (3)
C4—C3—C2	119.2 (3)	C10—C15—H15	119.5
C3—C4—C5	120.7 (3)	C14—C15—H15	119.5
C3—C4—H4	119.7	O7—C16—H16A	109.5
C5—C4—H4	119.7	O7—C16—H16B	109.5
C6—C5—C4	120.9 (3)	H16A—C16—H16B	109.5
C6—C5—H5	119.5	O7—C16—H16C	109.5
C4—C5—H5	119.5	H16A—C16—H16C	109.5
C5—C6—C1	119.5 (3)	H16B—C16—H16C	109.5
C5—C6—H6	120.3		
